# Perceived Value and Purchase Intention for Private-Label Dairy Products in Peru: The Mediating Role of Consumer Trust

**DOI:** 10.3390/foods15203713

**Published:** 2026-10-19

**Authors:** Edy Fernando Queque-Luque, Angeles Eden Arellan-Jaramillo, Jhudica Katherin Campos-Huaranca, Lorena Chino-Luque, Rosa Elvira Paredes-Cardenas, Luis Alberto Geraldo-Campos

**Affiliations:** Facultad de Administración y Negocios, Universidad Tecnológica del Perú, Lima 15046, Peru; c25922@utp.edu.pe (E.F.Q.-L.); u24223499@utp.edu.pe (A.E.A.-J.); u24243509@utp.edu.pe (J.K.C.-H.); u24269702@utp.edu.pe (L.C.-L.); u24272002@utp.edu.pe (R.E.P.-C.)

**Keywords:** perceived value, consumer trust, purchase intention, private label, dairy products, PLS-SEM

## Abstract

This study examined how five dimensions of perceived value—perceived quality, eco-nomic-financial value, functional-sensory value, emotional value, and social value—were associated with consumer trust and purchase intention for Tottus private-label dairy products in East Lima, Peru, including indirect statistical associations through trust. A cross-sectional survey was completed by 296 adult consumers with prior self-reported purchase or consumption of the studied private-label dairy products. The 37-item instrument was analyzed using PLS-SEM with 5000 bootstrap sub-samples, PLSpredict, and CVPAT. Perceived quality (β = 0.257), functional-sensory value (β = 0.168), emotional value (β = 0.277), and social value (β = 0.225) were positively as-sociated with consumer trust, whereas economic-financial value was not (β = 0.055, *p* = 0.202). Consumer trust was positively associated with purchase intention (β = 0.410, *p* < 0.001). Significant indirect associations through trust were observed for perceived quality (βind = 0.105), functional-sensory value (βind = 0.069), emotional value (βind = 0.114), and social value (βind = 0.092), but not economic-financial value (βind = 0.023, *p* = 0.218). The model explained 71.0% of the variance in trust and 73.3% in purchase intention, while PLSpredict/CVPAT supported out-of-sample predictive relevance for both endogenous constructs. The contribution is incremental and context specific: the findings show heterogeneous value–trust–intention association patterns within a Peruvian private-label dairy setting rather than a uniform perceived-value mechanism.

## 1. Introduction

Private-label brands are no longer competing solely on price; they are now used as tools for differentiation and retailer retention. In the food sector, this depends on the relationship between perceived quality, value for money, familiarity, risk, and trust [1,2]. Furthermore, continued purchasing after the initial trial requires that the experience confirms the product’s usefulness—not just that its price is lower than that of manufacturer brands [3].

Dairy products pose a significant challenge for these brands because they combine experiential attributes—such as taste, texture, and performance—with credibility attributes linked to safety, origin, and quality control; this finding is consistent with a review of 123 studies on dairy products and plant-based alternatives, which identified heterogeneous preferences regarding safety, origin, brand, and sensory characteristics, with limited evidence from South America [4]. Furthermore, familiarity and expectations are related to the evaluation of dairy products and their alternatives [5,6]; thus, the sensory quality of private-label dairy products can improve, although the differences depend on the category [7].

The literature indicates that an overall perceived-value score can mask heterogeneity across value dimensions. Benefit–sacrifice logic, consumer value theory, and the Perceived Value Scale (PERVAL) distinguish among quality, economic, functional, emotional, and social benefits [8,9,10]. Recent evidence likewise shows that these components need not display the same associations with brand image, loyalty, or purchase intention [11,12,13,14].

In food purchasing, trust is relevant because product quality, safety, origin, and future performance cannot always be fully verified before consumption [15]. Diverse value dimensions may therefore relate differently to trust and purchase intention: quality may signal dependability, economic value may reduce perceived financial sacrifice, functional-sensory value may reflect immediate product performance, and emotional and social value may capture distinct affective and relational evaluations [16,17]. This provides a theoretical basis for examining dimension-specific direct and indirect associations rather than assuming a single pathway.

In Latin America, evidence on private-label brands, healthy foods, and perceived value remains fragmented across product categories and countries [11,18,19]. In particular, less is known about how perceived quality, economic-financial value, functional-sensory value, emotional value, and social value coexist as correlates of consumer trust and purchase intention for private-label dairy products. The present study addresses this narrower gap among consumers in East Lima, Peru, by disaggregating five value dimensions, examining their direct and indirect statistical associations through consumer trust, and complementing explanatory assessment with out-of-sample prediction. The novelty is therefore incremental and contextual rather than based on the multidimensionality of perceived value itself.

This study is structured as follows: Section 2 focuses on establishing the study’s foundations and hypotheses. Section 3 describes the methodological approach adopted, as well as the characteristics of the participants, the scales, the ethical considerations, and the statistical techniques used. Section 4 presents the main results obtained, which address the hypotheses established in Section 2. Finally, Section 5 and Section 6 present the discussion and conclusions derived from the results.

## 2. Theoretical Foundations

### 2.1. Perceived Value, Consumer Trust, and Purchase Intention

Perceived value reflects consumers’ evaluation of benefits relative to the monetary, functional, and psychological sacrifices involved in an exchange [8]. Consumer value theory and PERVAL extend this logic by distinguishing among several sources of value whose relevance may vary across products and purchase situations [9,10]. For private-label foods, such differentiation is important because a product may be judged favorably on price, sensory performance, affective response, or social acceptance while still being evaluated separately in terms of trust and future purchase intention.

Perceived quality refers to an overall judgment of excellence and consistency; economic-financial value reflects affordability and value for money; functional-sensory value captures utility, taste, texture, and consumption performance; emotional value reflects enjoyment and positive affect; and social value reflects perceived approval, acceptance, and social identification. Reviews of private-label purchasing show that price, quality, familiarity, risk, and trust are all relevant to consumer decisions [1], while recent studies indicate that individual value dimensions may display different associations with brand image, loyalty, and willingness to consume food brands [11,12,20].

Consumer trust is defined here as the expectation that Tottus private-label dairy products and the associated brand will perform reliably, consistently, and safely. In food contexts, this expectation is relevant because quality, safety, and origin cannot always be fully verified before purchase [15]. Purchase intention, by contrast, represents a future-oriented behavioral disposition involving choice, repurchase, preference, and recommendation rather than observed purchasing behavior. Prior value–trust–intention models support examining whether product evaluations are associated with purchase intention directly and indirectly through trust [16,17].

### 2.2. Dimensions of Perceived Value and Consumer Trust

Perceived quality may be associated with consumer trust because judgments of freshness, consistency, and product excellence can function as cues when buyers cannot fully verify food quality or safety before consumption [15]. However, direct empirical support for a quality–trust path in comparable private-label dairy settings remains limited. García-Salirrosas et al. [19] support the conceptual distinction among quality, trust, and purchase intention rather than providing direct evidence for this specific path, while evidence from food-quality and safety research indicates that quality-related cues may be relevant to purchase decisions [21]. Private-label dairy research also shows that sensory-quality control can improve the competitive evaluation of retailer-owned products [7].

Economic-financial value concerns the relationship between benefits received and monetary sacrifice. Its association with trust is not necessarily positive because favorable price evaluations and reliability judgments are conceptually distinct. Price sensitivity is relevant to choices among differentiated dairy products [22,23], whereas Watanabe et al. [16] found no significant association between economic value and trust in an organic-food context.

Functional-sensory value reflects the product’s utility and sensory performance during consumption. Taste, texture, suitability, and functional performance are salient in the evaluation of dairy products and alternatives, and prior studies consistently identified sensory acceptability, and familiarity as relevant food-choice attributes [5,24,25]. These evaluations may therefore be associated with both perceived dependability and purchase intention.

Emotional and social value were specified separately because they capture conceptually different evaluations. Emotional value concerns enjoyment and positive affect, whereas social value concerns perceived acceptance, approval, and identity within a social environment. Watanabe et al. [16] found emotional value to be positively related to trust but did not find the same relationship for social value, and other food studies show that personal values, social norms, desire, and identity can relate differently to attitudes and intentions [26,27,28]. Based on this rationale, the following hypotheses were specified:

**H1a.**  *Perceived quality is positively associated with consumer trust.*

**H1b.**  *Economic-financial value is positively associated with consumer trust.*

**H1c.**  *Functional-sensory value is positively associated with consumer trust.*

**H1d.**  *Emotional value is positively associated with consumer trust.*

**H1e.**  *Social value is positively associated with consumer trust.*

### 2.3. Dimensions of Perceived Value and Purchase Intention

The dimensions of perceived value may also show distinct direct associations with purchase intention. Perceived quality can reduce uncertainty and strengthen product evaluation, although its behavioral relevance may depend on other signals such as trust [1,19].

Economic-financial value can reduce perceived monetary sacrifice, whereas functional-sensory value captures immediate utility and consumption performance. Emotional value can provide affective reasons for choice, while social value can reflect perceived approval or identification. Studies on dairy products, private-label brands, and sustainable foods support the relevance of these dimensions, but also indicate that their associations with purchase intention vary across product categories and contexts [3,29,30]. Based on the above, the following hypotheses were specified:

**H2a.**  *Perceived quality is positively associated with purchase intention.*

**H2b.**  *Economic-financial value is positively associated with purchase intention.*

**H2c.**  *Functional-sensory value is positively associated with purchase intention.*

**H2d.**  *Emotional value is positively associated with purchase intention.*

**H2e.**  *Social value is positively associated with purchase intention.*

### 2.4. Consumer Trust and Purchase Intent

Trust can reduce perceived vulnerability when buyers cannot fully evaluate a product’s quality, safety, or future performance before consumption. Wu et al. [15] showed that food trust may refer to the product, brand, supply-chain actors, and regulatory institutions. In the present study, the construct was deliberately anchored to Tottus private-label dairy products and the associated brand, rather than to trust in the retailer’s entire assortment or in external institutions.

Empirical evidence has demonstrated an association between trust, the adoption of private-label brands, and the intention to purchase fresh foods [2,17]. However, the results are not consistent, as they contradict the findings of Watanabe et al. [16], who did not find a significant direct path from trust to purchase intention in their sample of organic food consumers.

Brand trust has been reported as an intervening variable between corporate or advertising evaluations and purchase intention [31,32]. However, trust is not uniformly sufficient for purchase intention [33], and research on packaged-food technology adoption also reported non-significant trust-related paths [34]. The mixed evidence therefore supports testing, rather than assuming, the trust–purchase-intention relationship:

**H3.**  *Consumer trust is positively associated with purchase intent.*

### 2.5. The Mediating Role of Consumer Trust

In the context of fresh foods, Ling et al. [17] identified a value–trust–purchase-intention sequence. Other studies have modeled brand trust as an intervening variable between corporate or advertising evaluations and food purchase intention [31,32]. However, the evidence is not conclusive; Watanabe et al. [16] found that functional and emotional values were related to trust, whereas the trust–purchase-intention path was not significant.

**Figure 1:**
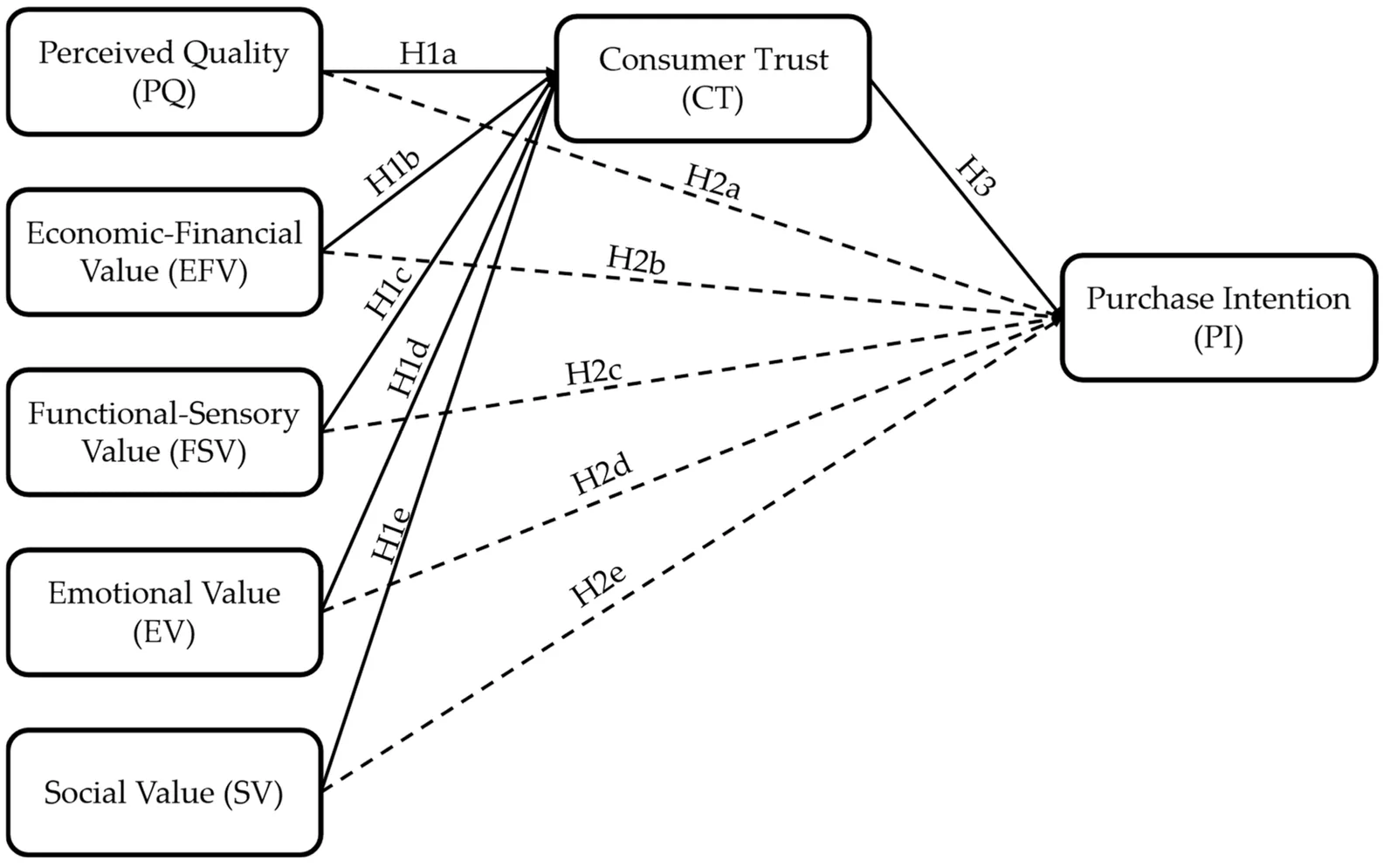
Conceptual model and hypotheses. Note: H4a–H4e denote indirect statistical associations through consumer trust; solid lines represent antecedents of trust and the trust–purchase-intention relationship; dashed lines represent direct associations between value dimensions and purchase intention.

Taken together, prior research provides a basis for examining whether each value dimension has an indirect statistical association with purchase intention through consumer trust [16,17,31,32]. Because the present design is cross-sectional, these hypotheses (Figure 1) concern indirect associations rather than temporal or causal mediation. The five dimensions were therefore tested separately:

**H4a.**  *Perceived quality has a positive indirect association with purchase intention through consumer trust.*

**H4b.**  *Economic-financial value has a positive indirect association with purchase intention through consumer trust.*

**H4c.**  *Functional-sensory value has a positive indirect association with purchase intention through consumer trust.*

**H4d.**  *Emotional value has a positive indirect association with purchase intention through consumer trust.*

**H4e.**  *Social value has a positive indirect association with purchase intention through consumer trust.*

## 3. Materials and Methods

### 3.1. Design and Unit of Analysis

This study used a quantitative, non-experimental, cross-sectional design with an explanatory-predictive scope. The unit of analysis was the adult consumer, whereas the object of evaluation was Tottus private-label dairy product portfolio, considered in aggregate rather than by individual dairy category. Throughout the questionnaire, the expression “Tottus dairy products” referred to dairy products marketed under the Tottus private label, not to third-party dairy brands sold by the retailer. The use of PLS-SEM enabled simultaneous estimation of multiple antecedents, indirect statistical associations, and out-of-sample predictive performance [35,36].

### 3.2. Measures

The measures were based on previously developed scales and food-consumption instruments [10,11,16,17,19,37]. The questionnaire was administered in Spanish and contained 37 items rated on a 5-point Likert scale. Eighteen items assessed five first-order perceived-value dimensions: perceived quality (PQ), economic-financial value (EFV), functional-sensory value (FSV), emotional value (EV), and social value (SV). Ten items assessed consumer trust (CT), and nine assessed purchase intention (PI). The documented adaptation process focused on contextualizing wording to Tottus private-label dairy products while preserving the intended construct content. No independent back-translation procedure is documented; therefore, the adaptation is reported as a contextual language adaptation rather than as a formal cross-language validation. The complete item wording, construct allocation, trust subdimensions, and construct-level source basis are reported in Supplementary Table S9.

As a preliminary adaptation step, five semi-structured instrumental interviews were conducted with consumers familiar with Tottus private-label dairy products and traditional brands. These interviews were used only to identify wording, comprehension, and contextual-relevance issues; they were not treated as a qualitative study and did not generate thematic findings. The resulting wording was then reviewed by three faculty researchers for clarity, conceptual consistency, and contextual appropriateness.

A separate pilot survey with 45 participants was subsequently used to verify item comprehensibility, survey flow, and operational functionality. Pilot cases were excluded from the final analytical sample. The pilot supported the usability of the adapted questionnaire but was not treated as independent evidence of construct validity; the final measurement properties were evaluated in the study sample.

The 18 perceived-value items were specified as PQ (PV1–PV4), EFV (PV5–PV8), FSV (PV9–PV12), EV (PV13–PV15), and SV (PV16–PV18). Consumer trust retained its 10 indicators and purchase intention retained its 9 indicators in the primary model. Item-retention decisions were evaluated through sensitivity analyses rather than by deleting indicators solely on the basis of a loading threshold; the corresponding robustness results are reported in Supplementary Tables S4 and S5.

Functional-sensory value was retained as an a priori, context-specific adapted domain covering household utility, consumption suitability, and sensory performance (PV9–PV12); it is not presented as a canonical PERVAL dimension. By contrast, emotional and social value were modeled separately in the revised specification because their item content and theoretical bases are distinct. For consumer trust, CC1–CC3 represented credibility, CC4–CC6 reliability, and CC7–CC10 perceived safety in the higher-order robustness model. All trust items were anchored to the Tottus private-label dairy product line or its associated brand; however, because the Tottus name simultaneously identifies the retailer and the private label, complete separation of product-line trust from retailer-level brand associations cannot be guaranteed. A separate sensory latent factor was not estimated because the instrument was not designed with multiple standalone sensory indicators; splitting the domain post hoc would therefore have produced an inadequately measured construct and changed the original measurement design.

### 3.3. Participants and Data Collection

Eligible participants were adults residing in East Lima, Peru—particularly in the district of Ate—who frequently purchased groceries for their households and who had self-reported having purchased or consumed Tottus private-label dairy products during the previous three months. Recruitment and survey completion took place outside the same Tottus supermarket in East Lima between 21–27 July 2026. Participants completed the questionnaire through Google Forms under supervised in-person administration using non-probability convenience sampling. Eligibility was based on participant self-report; no receipt or transaction-record verification was used.

A total of 365 individuals initiated the survey. Of these, 331 provided responses to all 37 substantive items, and 297 met the informed-consent and eligibility criteria. The intersection of completeness and eligibility yielded 296 cases for the final analyses. The complete data-verification flow is reported in Supplementary Table S1.

The participants’ characteristics show that 72.6% are women and 31.4% are in the 26 to 35-year-old age group. Regarding shopping habits, the most common frequency was every two weeks (30.4%), and the supermarket was identified as the primary shopping channel (50.3%). The full distribution of participant characteristics is reported in Supplementary Table S2.

### 3.4. Ethical Considerations

The initial protocol underwent an institutional review of ethical relevance on 1 June 2026. Based on the institutional ethics committee’s procedural guidelines, the review determined that the study did not require a full or expedited ethical review, as the activities were anonymous, non-sensitive, and targeted at an adult population. The responsible institution and the procedure followed are detailed in the non-blinded documentation. Electronic informed consent was obtained prior to administering the survey, during which participants were informed that participation was voluntary and they could exit the form at any time, and they were assured that no direct identifiers that would enable their identification were collected.

### 3.5. Data Preparation and Analysis

Preliminary analyses were conducted using JASP version 0.97.1. Common-method-related variance was examined using complementary diagnostics. Harman’s one-factor procedure was treated as a limited preliminary check [38]. Full-collinearity VIFs were then calculated from latent-variable scores following the full-collinearity approach [39], and a single-factor CFA was compared descriptively with the theoretically specified seven-factor CFA. These procedures were interpreted jointly and not as definitive proof of the absence of common-method variance. Multivariate normality was also assessed using Mardia’s test.

The measurement structure was additionally examined using CFA with the same 37 indicators. The theoretically specified seven-factor solution was compared with a single-factor alternative as a complementary common-method diagnostic. In addition, consumer trust was re-estimated in PLS-SEM as a reflective–reflective higher-order construct composed of credibility, reliability, and perceived safety using a two-stage approach; this specification was treated as a robustness analysis rather than as the primary structural model.

The structural model was estimated using SmartPLS 4 [40] with a path-weighting scheme, standardized results, a maximum of 3000 iterations, and a stopping criterion of 10^−7^. Reliability, average variance extracted, HTMT, and collinearity were evaluated [35,36,41]. Path coefficients and indirect associations were assessed with 5000 bootstrap subsamples and 95% percentile confidence intervals; R^2^, adjusted R^2^, f^2^, total effects, and internal VIF were also calculated. Descriptive labels for indirect-only and complementary indirect patterns followed the logic proposed by Zhao et al. [42], but, because the design was cross-sectional, these were interpreted as statistical association patterns rather than causal mediation. Sensitivity analyses examined reduced PI specifications and the higher-order CT specification. Predictive performance was evaluated using PLSpredict with 10 folds, 10 repetitions, and a fixed seed, together with CVPAT, comparing Q^2^predict and prediction errors against a linear-model benchmark and predictive loss against both the indicator-average and linear-model benchmarks [43,44].

## 4. Results

### 4.1. Common-Method Assessment and Sensitivity Analyses

Common-method-related variance was evaluated using complementary diagnostics rather than relying on a single procedure. Full-collinearity VIFs calculated from latent-variable scores ranged from 2.008 to 4.082. PQ (2.551), EFV (2.008), FSV (3.247), and SV (2.370) remained below the conservative 3.3 benchmark, whereas EV (3.436), CT (4.082), and PI (3.745) exceeded it. Accordingly, common-method variance could not be completely ruled out on the basis of this diagnostic alone. A complementary CFA comparison nevertheless favored the theoretically specified seven-factor measurement structure, χ^2^(601) = 986.00, CFI = 0.996, TLI = 0.996, RMSEA = 0.047, 90% CI [0.041, 0.052], SRMR = 0.055, over a single-factor model, χ^2^(628) = 1936.00, CFI = 0.987, TLI = 0.987, RMSEA = 0.084, 90% CI [0.080, 0.088], SRMR = 0.071. Thus, the covariance structure was not adequately represented as a single common factor, although residual common-method variance cannot be excluded. The complete full-collinearity and CFA comparison is reported in Supplementary Table S6.

Purchase intention was examined under three reduced specifications as sensitivity analyses: excluding PI_P3, excluding PI_R1, and excluding both items simultaneously. The structural paths and indirect associations remained substantively stable across all specifications. None of the reduced models improved discriminant validity. Relative to the complete PI specification (α = 0.893, ρA = 0.899, ρC = 0.914, AVE = 0.542, R^2^PI = 0.733, HTMTPI–CT = 0.884), the simultaneous exclusion of both items reduced reliability and explained variance (α = 0.858, ρA = 0.868, ρC = 0.892, AVE = 0.544, R^2^PI = 0.725) and increased HTMTPI–CT to 0.896. The complete nine-item PI specification was therefore retained. Detailed comparisons are provided in Supplementary Table S4.

Consumer trust was also re-estimated as a reflective–reflective higher-order construct composed of credibility, reliability, and perceived safety using a two-stage approach. The three lower-order dimensions loaded strongly on the higher-order construct (λ = 0.890–0.897, all *p* < 0.001), and the structural results remained substantively unchanged. However, the higher-order specification increased the CT–PI HTMT value to 0.909 (95% CI [0.858, 0.951]), compared with 0.884 in the first-order specification. Therefore, the first-order ten-item CT construct was retained as the primary specification, while the hierarchical model was treated as a robustness analysis. The higher-order robustness results are summarized in Supplementary Table S5.

### 4.2. PLS-SEM Measurement Model

The primary measurement model retained all 37 indicators across seven reflective constructs. Outer loadings ranged from 0.624 to 0.920 and were significant at *p* < 0.001. Three indicators were below 0.70—CT_C1 (λ = 0.696), PI_F1 (λ = 0.624), and PI_R2 (λ = 0.651)—but were retained because the corresponding constructs showed adequate internal consistency and convergent validity and because the sensitivity analyses did not support item deletion. The remaining loading ranges were PQ = 0.792–0.871, EFV = 0.748–0.845, FSV = 0.804–0.885, EV = 0.864–0.920, SV = 0.826–0.850, CT = 0.696–0.805, and PI = 0.624–0.807.

Internal consistency and convergent validity were satisfactory for all constructs: Cronbach’s α ranged from 0.792 to 0.918, ρA from 0.795 to 0.919, ρC from 0.865 to 0.932, and AVE from 0.542 to 0.796 (Table 1). HTMT values ranged from 0.656 to 0.899. All point estimates were below 0.90, although the conservative 0.85 criterion flagged EV–CT (0.854), FSV–EV (0.899), and PI–CT (0.884) as pairs with residual conceptual proximity. Bootstrap HTMT inference did not indicate empirical indistinguishability because none of the 95% intervals included 1; the corresponding intervals were [0.797, 0.901], [0.831, 0.956], and [0.833, 0.926], respectively. Discriminant validity was therefore considered acceptable under the 0.90 and inferential criteria, while the conservative 0.85 assessment was interpreted cautiously rather than as unequivocal evidence of separation. The complete HTMT matrix and bootstrap confidence intervals are provided in Supplementary Table S3.

### 4.3. Structural Model and Indirect Associations

Inner VIF values ranged from 1.897 to 3.448, remaining below the conventional threshold of 5. The model explained 71.0% of the variance in consumer trust (R^2^ = 0.710, adjusted R^2^ = 0.705) and 73.3% of the variance in purchase intention (R^2^ = 0.733, adjusted R^2^ = 0.727). For consumer trust, perceived quality (β = 0.257, *p* < 0.001, 95% CI [0.146, 0.377]), functional-sensory value (β = 0.168, *p* = 0.010, 95% CI [0.044, 0.294]), emotional value (β = 0.277, *p* < 0.001, 95% CI [0.162, 0.391]), and social value (β = 0.225, *p* < 0.001, 95% CI [0.123, 0.322]) were positively associated with CT, whereas economic-financial value was not (β = 0.055, *p* = 0.202, 95% CI [−0.033, 0.142]).

Purchase intention was positively associated with consumer trust (β = 0.410, *p* < 0.001, 95% CI [0.273, 0.547]), economic-financial value (β = 0.164, *p* < 0.001, 95% CI [0.074, 0.257]), functional-sensory value (β = 0.163, *p* = 0.009, 95% CI [0.039, 0.281]), and social value (β = 0.139, *p* = 0.007, 95% CI [0.035, 0.236]). Emotional value did not show a direct association with PI (β = 0.018, *p* = 0.781, 95% CI [−0.105, 0.151]). The PQ → PI coefficient was nominally significant (β = 0.098, *p* = 0.049), but its percentile 95% CI included zero [−0.003, 0.190]; consequently, this direct association was treated as borderline and not as robust support for a direct relationship.

The indirect associations through CT were significant for perceived quality (βind = 0.105, *p* = 0.001, 95% CI [0.053, 0.174]), functional-sensory value (βind = 0.069, *p* = 0.020, 95% CI [0.017, 0.132]), emotional value (βind = 0.114, *p* < 0.001, 95% CI [0.061, 0.174]), and social value (βind = 0.092, *p* = 0.001, 95% CI [0.044, 0.151]), but not for economic-financial value (βind = 0.023, *p* = 0.218, 95% CI [−0.014, 0.060]). Given the cross-sectional design, these results are interpreted as indirect statistical association patterns rather than causal mediation. The FSV and SV results were consistent with complementary indirect patterns, EV with an indirect-only pattern, and PQ with a robust indirect association accompanied by a borderline direct coefficient. The direct associations, indirect associations, effect sizes, confidence intervals, and hypothesis decisions are summarized in Table 2.

### 4.4. Out-of-Sample Predictive Performance

PLSpredict was estimated using 10-fold cross-validation with 10 repetitions and a fixed seed. All 19 endogenous indicators showed positive Q^2^predict values, ranging from 0.238 to 0.513. At the latent-variable level, Q^2^predict was 0.699 for CT and 0.669 for PI. Using RMSE as the primary prediction-error criterion, PLS-SEM produced lower errors than the linear-model benchmark for 17 of the 19 endogenous indicators; CT_R1 was virtually identical across approaches (0.573 vs. 0.573), and CT_C1 showed a slightly higher PLS-SEM RMSE (0.587 vs. 0.570). These results indicate substantial out-of-sample predictive performance. Indicator-level predictive results are reported in Supplementary Table S7.

CVPAT provided convergent evidence. Relative to the indicator-average benchmark, PLS-SEM showed significantly lower prediction loss for CT (ΔLoss = −0.209, t = 7.904, *p* < 0.001), PI (ΔLoss = −0.216, t = 7.835, *p* < 0.001), and the overall model (ΔLoss = −0.213, t = 8.456, *p* < 0.001). Relative to the linear-model benchmark, prediction loss was also significantly lower for CT (ΔLoss = −0.013, t = 2.347, *p* = 0.020), PI (ΔLoss = −0.028, t = 3.585, *p* < 0.001), and the overall model (ΔLoss = −0.020, t = 4.280, *p* < 0.001). Thus, both PLSpredict and CVPAT supported the predictive relevance of the final model. Latent-level PLSpredict and CVPAT results are summarized in Supplementary Table S8.

## 5. Discussion

This study examined how five dimensions of perceived value—perceived quality, economic-financial value, functional-sensory value, emotional value, and social value—were associated with consumer trust and purchase intention for Tottus private-label dairy products. The findings show that these value dimensions were not related to trust and purchase intention in a uniform way. Perceived quality, functional-sensory value, emotional value, and social value were positively associated with consumer trust, whereas economic-financial value was not. Purchase intention, in turn, was positively associated with consumer trust, economic-financial value, functional-sensory value, and social value; emotional value showed no direct association, and the direct association between perceived quality and purchase intention was borderline because its percentile confidence interval included zero. This pattern supports interpreting perceived value dimension by dimension rather than as a single undifferentiated score, which is consistent with recent evidence showing that the components of perceived value can differ in their behavioral relevance across food contexts [12,14].

Perceived quality was positively associated with consumer trust and showed a robust indirect association with purchase intention through trust, while its direct relationship with purchase intention was not robust. This suggests that, in this sample, quality evaluations were more closely connected with expectations of reliability than with an immediate behavioral disposition to purchase. Such an interpretation is consistent with the broader private-label and food literature in which quality, risk, brand cues, and trust are conceptually distinct components of consumer evaluation [1,19]. García-Salirrosas et al. [19], in particular, provided contextual evidence for the distinction between perceived quality and trust rather than direct evidence for a quality–trust path. Recent private-label research in Spain likewise showed that perceived product quality remains closely associated with loyalty and category engagement as retailer-owned brands compete on quality and perceived value rather than price alone [45]. This evidence strengthens the relevance of quality in private-label evaluation, but it does not establish that quality directly produces trust. Accordingly, the present result is best interpreted as a robust indirect association through trust rather than as evidence that perceived quality itself is equivalent to overall value or necessarily translates directly into purchase intention.

Economic-financial value displayed a different pattern: it was positively associated with purchase intention but not with consumer trust, and its indirect association through trust was not significant. This result is consistent with a benefit–sacrifice interpretation of value, whereby price affordability and value for money may reduce the perceived financial sacrifice of choosing the product without necessarily changing beliefs about its reliability or safety. Evidence from private-label and differentiated-food markets similarly indicates that price sensitivity and value-for-money considerations coexist with quality and risk evaluations rather than replacing them [18,23]. Watanabe et al. [16] also reported no significant association between economic value and trust. Moreover, recent Latin American evidence found that economic value did not significantly predict willingness to consume healthy food brands, whereas other value dimensions did [12]. The contrast with the current positive association between EFV and PI suggests that the behavioral relevance of monetary value may vary depending on product positioning and the consumption context; this interpretation should therefore be regarded as context specific rather than universal.

Functional-sensory value was positively associated with both consumer trust and purchase intention, and its indirect association through trust was also significant. This dual pattern is theoretically plausible because functional and sensory evaluations capture both immediate consumption utility and evidence about whether the product performs as expected. Taste, texture, utility, and suitability for consumption can therefore contribute directly to willingness to choose the product while also informing expectations of reliability. Previous studies on dairy products and food alternatives emphasized the importance of sensory acceptability, familiarity, and product performance in consumer evaluation [5,24]. More recent dairy-specific evidence also showed that flavor, origin, brand, and price are considered important purchase attributes and that sensory characteristics remain central to consumer appraisal of milk products [46]. These studies do not demonstrate the trust pathway estimated here, but they reinforce the substantive importance of functional and sensory cues in dairy-product evaluation. The present combination of direct and indirect associations therefore supports interpreting functional-sensory value as relevant to both immediate utility and trust-related evaluation.

Separating emotional value from social value produced two distinct patterns that were obscured in the original combined specification. Emotional value was positively associated with consumer trust but not directly with purchase intention, whereas social value was positively associated with both trust and purchase intention. This distinction is supported by the heterogeneity observed in recent food-consumption research. García-Salirrosas et al. [12] found both emotional and social value to be relevant to willingness to consume healthy food brands, whereas Clottey et al. [47] reported a significant relationship between social value and purchase intention for waste-to-value foods but no significant relationship for emotional value. The latter pattern resembles the direct associations observed in the present study. Watanabe et al. [16] also found emotional value to be positively related to trust. Taken together, these results reinforce the decision to model emotional and social value separately: their behavioral relevance appears to vary across food categories and consumption contexts rather than operating as interchangeable components of a single construct.

Consumer trust showed a positive association with purchase intention, supporting H3 and the value–trust–intention logic used in the conceptual model. This finding is consistent with research in fresh-food and sustainability contexts in which brand trust transmits favorable product or corporate evaluations to purchase intention [17,31,32]. It also fits recent evidence showing that trust in food-chain actors can strengthen the translation of consumer motivations into intentions for sustainable and novel foods, although the magnitude and relevance of this role differ by type of food choice and actor [48]. Recent private-label research further indicates that credible private-label images and lower perceived risk are linked to more favorable attitudes and purchase intentions in food retail [49]. At the same time, the present result differs from Watanabe et al. [16], who found no significant direct trust–purchase-intention relationship among Brazilian organic-food consumers and from studies showing that trust alone may be insufficient for purchase decisions in green private-label or technology-enabled packaged-food settings [33,34]. These differences reinforce a context-dependent interpretation of trust rather than a universal food-consumption mechanism.

The indirect-association results further clarify the role of consumer trust. Trust carried significant indirect associations from perceived quality, functional-sensory value, emotional value, and social value to purchase intention, but not from economic-financial value. The functional-sensory and social-value results showed significant direct and indirect associations in the same direction, whereas emotional value showed an indirect-only pattern. For perceived quality, the indirect association was robust, while the direct coefficient was borderline and its percentile confidence interval included zero. These patterns remained substantively stable when purchase intention was re-estimated after excluding the value-referent item, the recommendation item, or both items. The higher-order trust specification likewise reproduced the main structural conclusions, although it increased the HTMT ratio between trust and purchase intention relative to the first-order model. Together with the conservative HTMT flags observed for some construct pairs, this supports a cautious interpretation: the structural pattern is robust, but residual conceptual proximity remains. Because the data are cross-sectional, these findings describe indirect statistical association patterns and should not be interpreted as evidence of temporal or causal mediation.

The theoretical contribution of the study is incremental and context specific. Multidimensional perceived value is already well established [8,9,10,11,12]; the contribution here lies in showing, within a Peruvian private-label dairy setting, that five value dimensions are associated with trust and purchase intention through different direct and indirect patterns. Economic-financial value was associated mainly with purchase intention, emotional value mainly with trust and an indirect association with purchase intention, and functional-sensory and social value showed both direct and trust-related associations. Perceived quality was principally related to purchase intention through trust. Recent private-label research similarly emphasized that retailer-owned brands increasingly compete through combinations of perceived quality, value, risk reduction, and brand-related evaluations rather than through price alone [45,49]. The present study extends that literature by showing heterogeneity across specific value dimensions and by examining trust as an intervening statistical association within a private-label dairy portfolio in Peru, rather than by presenting multidimensional value itself as novel. The explanatory and predictive evidence also converged: the model explained substantial variance in consumer trust and purchase intention, all endogenous indicators showed positive Q^2^predict values, and CVPAT indicated significantly lower prediction loss than both the indicator-average and linear-model benchmarks for consumer trust, purchase intention, and the overall model. Accordingly, the final specification supports out-of-sample predictive relevance for both endogenous constructs, while these predictive results complement, but do not convert, the cross-sectional associations into causal evidence [43,44].

## 6. Conclusions

Purchase intention for Tottus private-label dairy products was associated with perceived value through heterogeneous pathways rather than through a single overall value mechanism. Perceived quality was principally related to purchase intention through consumer trust; economic-financial value showed a direct association with purchase intention but no significant indirect association through trust; functional-sensory and social value showed both direct and significant indirect associations; and emotional value was associated with purchase intention indirectly through trust rather than directly. Consumer trust itself was positively associated with purchase intention. These patterns remained substantively stable across the sensitivity analyses of purchase intention and the alternative higher-order specification of consumer trust. The model also demonstrated out-of-sample predictive relevance for both consumer trust and purchase intention. Because the study was cross-sectional, these findings should be interpreted as direct and indirect statistical associations rather than causal effects.

### 6.1. Practical Implications

The findings suggest several practical implications for managers of private-label dairy portfolios. These implications are limited to the value dimensions and trust perceptions measured in the study and should not be interpreted as evidence about unmeasured operational practices, manufacturing arrangements, or consumer segments.

First, perceived quality and functional-sensory value were both positively associated with consumer trust, while functional-sensory value was also directly associated with purchase intention. This pattern supports prioritizing the consistency of product attributes that consumers can evaluate, particularly sensory and performance-related cues such as flavor, texture, and perceived functionality. Recent private-label and dairy research likewise emphasized the role of perceived quality and sensory attributes in consumer evaluation [7,45,46]. The managerial implication is therefore to make quality and sensory consistency visible and verifiable at the point of evaluation, rather than assuming that a low price alone will sustain favorable purchase intentions.

Second, economic-financial value was associated with purchase intention but not with consumer trust. Accordingly, price and value-for-money positioning may contribute to purchase consideration without necessarily strengthening perceptions of reliability or safety. Managers should therefore avoid treating economic value as a substitute for quality- or trust-related signals. The data support distinguishing the communication of economic value from the communication of product dependability; they do not support a specific promotional format such as discounting, which was not examined in this study.

Third, emotional and social value should not be managed as interchangeable dimensions. Emotional value was associated with trust but not directly with purchase intention, whereas social value was associated with both trust and purchase intention. This suggests that communications intended to strengthen a positive effect toward the product and those intended to reinforce social acceptance may serve different purposes. However, the present study did not test specific communication formats, messages, or segmentation strategies; consequently, such interventions should be evaluated experimentally before being adopted as evidence-based recommendations.

### 6.2. Limitations and Future Research

Several limitations delimit the interpretation of the findings. First, the cross-sectional design does not establish temporal ordering or causality; therefore, the direct and indirect paths should be interpreted as associations. Convenience sampling and recruitment from consumers of a single retailer in one urban area also constrain external validity, and the sex distribution of the sample was unbalanced. In addition, purchase intention was self-reported rather than observed behavior, and the private-label dairy portfolio was evaluated in aggregate. The results may therefore differ across specific dairy categories, purchase occasions, retailers, or geographic settings.

Second, all focal constructs were measured in the same self-report survey, so common-method variance cannot be excluded (See Table S6). The complementary diagnostics did not support dominance by a single common factor: the theoretically specified seven-factor CFA fitted substantially better than the single-factor model. Nevertheless, full-collinearity VIFs exceeded the conservative 3.3 criterion for emotional value, consumer trust, and purchase intention. These mixed diagnostics warrant caution. Future studies should incorporate procedural remedies at the design stage, such as temporal or source separation where feasible, and may include an appropriate marker variable or other method-factor approaches when theoretically justified.

Third, although the revised seven-factor specification separated emotional and social value and all HTMT point estimates were below 0.90, several pairs exceeded the more conservative 0.85 criterion, particularly functional-sensory value with emotional value and consumer trust with purchase intention. Bootstrap HTMT intervals excluded 1.00, but this does not eliminate the possibility of conceptual proximity. The higher-order consumer-trust robustness model reproduced the structural conclusions but increased the CT–PI HTMT value. Accordingly, the first-order trust specification was retained as the primary model, and the discriminant-validity evidence should be regarded as acceptable under the 0.90 and inferential criteria but not unequivocal under the stricter 0.85 benchmark.

Fourth, purchase intention included future choice, preference, and recommendation indicators. The sensitivity analyses excluding the value-referent item, the recommendation item, and both items simultaneously yielded substantively stable structural conclusions and did not improve discriminant validity, supporting retention of the complete nine-item specification. Even so, future research could validate narrower behavioral-intention measures against actual purchase or repurchase data. The preliminary interviews, internal faculty review, and pilot survey supported contextualization and usability of the questionnaire but should not be interpreted as independent evidence of content validity beyond the adapted source scales.

Fifth, eligibility for prior private-label dairy purchase or consumption was based on participant self-report rather than receipt or transaction-record verification. Although the questionnaire was explicitly contextualized to Tottus private-label dairy products and several items contrasted them with other or traditional brands, the shared Tottus name may still allow some retailer-level brand associations to enter the trust responses, particularly for the credibility item referring to Tottus as a trustworthy brand. Future studies should verify product ownership or purchase more directly—for example, through product-package recognition, transaction records, or category-specific screening—and should distinguish retailer trust from private-label product trust when both are theoretically relevant.

Future research should prioritize longitudinal or experimental designs, probability-based or more diverse sampling frames, and behavioral purchase outcomes. Replication across retailers, regions, and individual dairy categories would clarify the boundary conditions of the observed associations. Multi-group or measurement-invariance analyses could test whether the model behaves similarly across demographic or shopping segments when adequately powered. Further work may also compare first-order and higher-order trust specifications, examine alternative theoretically justified measurement structures for closely related value dimensions, and use multi-source or temporally separated designs to assess the robustness of the value–trust–purchase-intention relationships.

## Figures and Tables

**Table 1:** Indicator loadings, reliability, and convergent validity of the primary measurement model.

Construct	Items	λ	α	ρA	ρC	AVE
Perceived Quality (PQ)	PQ1	0.822	0.838	0.839	0.892	0.673
	PQ2	0.792				
	PQ3	0.871				
	PQ4	0.794				
Economic-Financial Value (EFV)	EFV1	0.748	0.792	0.796	0.865	0.617
	EFV2	0.845				
	EFV3	0.759				
	EFV4	0.785				
Functional-Sensory Value (FSV)	FSV1	0.804	0.844	0.848	0.895	0.682
	FSV2	0.885				
	FSV3	0.804				
	FSV4	0.806				
Emotional Value (EV)	EV1	0.864	0.872	0.874	0.921	0.796
	EV2	0.920				
	EV3	0.891				
Social Value (SV)	SV1	0.850	0.792	0.795	0.878	0.706
	SV2	0.843				
	SV3	0.826				
Consumer Trust (CT)	CT_C1	0.696	0.918	0.919	0.932	0.577
	CT_C2	0.765				
	CT_C3	0.760				
	CT_R1	0.745				
	CT_R2	0.788				
	CT_R3	0.805				
	CT_S1	0.752				
	CT_S2	0.752				
	CT_S3	0.775				
	CT_S4	0.751				
Purchase Intention (PI)	PI_F1	0.624	0.893	0.899	0.914	0.542
	PI_F2	0.764				
	PI_F3	0.720				
	PI_P1	0.771				
	PI_P2	0.807				
	PI_P3	0.782				
	PI_R1	0.763				
	PI_R2	0.651				
	PI_R3	0.724				

Note. λ = standardized outer loading; α = Cronbach’s alpha; ρA and ρC = composite reliability; AVE = average variance extracted. Reliability and AVE are reported once per construct. All outer loadings were significant at *p* < 0.001. The complete HTMT matrix and bootstrap confidence intervals are reported in Supplementary Table S3.

**Table 2:** Direct associations, indirect associations, and hypothesis decisions.

Hypothesis	Path	β	*p*	95% CI	f^2^/Mediation	Decision
H1a	PQ → CT	0.257	<0.001	[0.146, 0.377]	f^2^ = 0.099	Supported
H1b	EFV → CT	0.055	0.202	[−0.033, 0.142]	f^2^ = 0.006	Not supported
H1c	FSV → CT	0.168	0.010	[0.044, 0.294]	f^2^ = 0.032	Supported
H1d	EV → CT	0.277	<0.001	[0.162, 0.391]	f^2^ = 0.083	Supported
H1e	SV → CT	0.225	<0.001	[0.123, 0.322]	f^2^ = 0.082	Supported
H2a	PQ → PI	0.098	0.049	[−0.003, 0.190]	f^2^ = 0.014	Not supported ^1^
H2b	EFV → PI	0.164	<0.001	[0.074, 0.257]	f^2^ = 0.053	Supported
H2c	FSV → PI	0.163	0.009	[0.039, 0.281]	f^2^ = 0.032	Supported
H2d	EV → PI	0.018	0.781	[−0.105, 0.151]	f^2^ = 0.000	Not supported
H2e	SV → PI	0.139	0.007	[0.035, 0.236]	f^2^ = 0.031	Supported
H3	CT → PI	0.410	<0.001	[0.273, 0.547]	f^2^ = 0.182	Supported
H4a	PQ → CT → PI	0.105	0.001	[0.053, 0.174]	Robust indirect; direct borderline	Supported
H4b	EFV → CT → PI	0.023	0.218	[−0.014, 0.060]	No significant indirect association	Not supported
H4c	FSV → CT → PI	0.069	0.020	[0.017, 0.132]	Complementary pattern	Supported
H4d	EV → CT → PI	0.114	<0.001	[0.061, 0.174]	Indirect-only pattern	Supported
H4e	SV → CT → PI	0.092	0.001	[0.044, 0.151]	Complementary pattern	Supported

Note. Two-tailed percentile bootstrap with 5000 subsamples. R^2^: CT = 0.710 (adjusted R^2^ = 0.705); PI = 0.733 (adjusted R^2^ = 0.727). Indirect-pattern labels are descriptive and do not imply causal mediation in this cross-sectional design. ^1^ The PQ → PI coefficient had *p* = 0.049, but its percentile 95% CI included zero; it was therefore not treated as robust support for H2a.

## Data Availability

The data supporting the findings of this study are available in the Open Science Framework (OSF) repository at: https://osf.io/k8xns/overview?view_only=c1ae741dea6f42f3b7cdc3e1999ad9fa (accessed on 21 September 2026).

## Supplementary Materials

The following supporting information can be downloaded at https://www.mdpi.com/article/10.3390/foods15203713/s1, Table S1: Flow of records leading to the analytical sample; Table S2: Participant characteristics; Table S3: HTMT ratios and bootstrap 95% confidence intervals; Table S4: Sensitivity analysis for alternative purchase-intention specifications; Table S5: Higher-order consumer-trust robustness analysis; Table S6: Complementary common-method diagnostics; Table S7: PLSpredict results at the endogenous-indicator level; Table S8: Latent-level PLSpredict and CVPAT results; Table S9: Questionnaire item wording, construct allocation, consumer-trust subdimensions, and construct-level source basis.

## Abbreviations

The following abbreviations are used in this manuscript:

PERVAL	Perceived Value Scale
PLS-SEM	Partial Least Squares Structural Equation Modeling
PQ	Perceived Quality
EFV	Economic-Financial Value
FSV	Functional-Sensory Value
EV	Emotional Value
SV	Social Value
CT	Consumer Trust
PI	Purchase Intention
CFA	Confirmatory Factor Analysis
HTMT	Heterotrait–Monotrait Ratio
VIF	Variance Inflation Factor
FCVIF	Full-Collinearity Variance Inflation Factor
CVPAT	Cross-Validated Predictive Ability Test
Q^2^predict	Predictive Relevance Statistic
RMSE	Root Mean Squared Error
MAE	Mean Absolute Error
RMSEA	Root Mean Square Error of Approximation
SRMR	Standardized Root Mean Square Residual
TLI	Tucker–Lewis Index
CFI	Comparative Fit Index
AVE	Average Variance Extracted
R^2^	Coefficient of Determination
IA	Indicator-Average Benchmark
LM	Linear-Model Benchmark
OSF	Open Science Framework
