# Supplementary material for: Perceived Value and Purchase Intention for Private-Label Dairy Products in Peru: The Mediating Role of Consumer Trust
Source: Foods. 2026 Oct 19;15(20):3713. doi: 10.3390/foods15203713 (PMC13704347; doi:10.3390/foods15203713)
Supplement: Supplementary file 1 [file foods-15-03713-s001.zip › foods-4611858-supplementary.pdf]

Supplementary Materials

Table S1. Flow of records leading to the analytical sample

Stage	n	Criterion
Forms initiated	365	Initial registration
Respondents with responses to the 37 substantive items	331	Questionnaire completeness
Respondents providing consent and meeting eligibility criteria	297	Ethical and eligibility screening
Complete and eligible cases	296	Intersection of the two previous filters

Note. Complete and eligible cases (n = 296) constituted the analytical sample used in the final models.

Table S2. Participant characteristics

Variable	Category	n	%
Age	18–25	62	20.9
Age	26–35	93	31.4
Age	36–45	61	20.6
Age	46–55	61	20.6
Age	56 years or older	19	6.4
Sex	Female	215	72.6
Sex	Male	75	25.3
Sex	Prefer not to answer	6	2.0
Purchase frequency	Once a week	38	12.8
Purchase frequency	Two or more times per week	52	17.6
Purchase frequency	Every two weeks	90	30.4
Purchase frequency	Once a month	76	25.7
Purchase frequency	Occasionally	40	13.5
Monthly spending on dairy products	Less than S/ 50	96	32.4
Monthly spending on dairy products	S/ 50–S/ 100	108	36.5
Monthly spending on dairy products	S/ 101–S/ 150	76	25.7
Monthly spending on dairy products	More than S/ 150	16	5.4
Main purchase channel	Supermarket	149	50.3
Main purchase channel	Traditional market	53	17.9
Main purchase channel	Neighborhood store	43	14.5
Main purchase channel	Convenience store	26	8.8
Main purchase channel	Online	25	8.4

Note. Percentages are calculated using the final analytical sample (N = 296); minor differences from 100% are due to rounding.

Table S3. HTMT ratios and bootstrap 95% confidence intervals

Construct pair	HTMT	95% CI lower	95% CI upper
EFV–CT	0.688	0.598	0.769
EV–CT	0.854	0.797	0.901
EV–EFV	0.656	0.561	0.743
FSV–CT	0.831	0.751	0.896
FSV–EFV	0.754	0.673	0.827
FSV–EV	0.899	0.831	0.956

PI–CT	0.884	0.833	0.926
PI–EFV	0.776	0.688	0.854
PI–EV	0.795	0.730	0.857
PI–FSV	0.836	0.772	0.894
PQ–CT	0.816	0.741	0.879
PQ–EFV	0.761	0.667	0.843
PQ–EV	0.786	0.704	0.854
PQ–FSV	0.795	0.712	0.866
PQ–PI	0.797	0.729	0.857
SV–CT	0.817	0.743	0.878
SV–EFV	0.660	0.542	0.767
SV–EV	0.830	0.761	0.896
SV–FSV	0.784	0.695	0.865
SV–PI	0.806	0.713	0.887
SV–PQ	0.684	0.570	0.786

Note. None of the bootstrap confidence intervals included 1.00. EV–CT, FSV–EV, and PI–CT exceeded the conservative .85 point-estimate criterion but remained below .90.

Table S4. Sensitivity analysis for alternative purchase-intention specifications

Model	PI specification	α	ϱA	ϱC	AVE	R^2 PI	HTMT PI–CT	SRMR
A (primary)	All 9 items	.893	.899	.914	.542	.733	.884	.060
B	Without PI_P3	.877	.884	.903	.540	.728	.890	.061
C	Without PI_R1	.879	.886	.904	.543	.733	.889	.060
D	Without PI_P3 and PI_R1	.858	.868	.892	.544	.725	.896	.061

Note. Structural and indirect-association conclusions were substantively stable across all four specifications. The complete nine-item PI specification was retained.

Table S5. Higher-order consumer-trust robustness analysis

Component	Estimate	p	95% CI	Interpretation
Credibility → CT	.890	< .001	[.866, .910]	Strong second-order loading
Reliability → CT	.890	< .001	[.865, .910]	Strong second-order loading
Perceived safety → CT	.897	< .001	[.867, .921]	Strong second-order loading
CT reliability	α=.872; ϱA=.872; ϱC=.921	—	AVE=.796	Adequate reliability/convergence
CT–PI HTMT	.909	—	[.858, .951]	Higher than primary first-order CT (.884)
R^2 CT / PI	.715 / .732	—	—	Structural conclusions stable

Note. The higher-order specification supported convergence of credibility, reliability, and perceived safety but did not improve CT–PI discriminant separation; therefore, the first-order CT specification was retained as primary.

Table S6. Complementary common-method diagnostics

Harman single-factor procedure	First unrotated factor	46.19% of variance	Preliminary heuristic only; not sufficient to rule out common-method variance
Full-collinearity VIF	PQ	2.551	Below 3.3
Full-collinearity VIF	EFV	2.008	Below 3.3
Full-collinearity VIF	FSV	3.247	Below 3.3; near threshold
Full-collinearity VIF	EV	3.436	Above conservative 3.3 criterion

Full-collinearity VIF	SV	2.370	Below 3.3
Full-collinearity VIF	CT	4.082	Above conservative 3.3 criterion
Full-collinearity VIF	PI	3.745	Above conservative 3.3 criterion
CFA	Seven-factor model	$\chi^2(601)$=986.00; CFI=.996; TLI=.996; RMSEA=.047; SRMR=.055	Good fit
CFA	Single-factor model	$\chi^2(628)$=1936.00; CFI=.987; TLI=.987; RMSEA=.084; SRMR=.071	Substantially poorer fit, particularly RMSEA/SRMR

Note. The combined diagnostics do not support dominance by a single common factor, although residual common-method variance cannot be completely excluded.

Table S7. PLSpredict results at the endogenous-indicator level

Indicator	Q^2predict	PLS-SEM RMSE	LM RMSE	PLS lower than LM?
CT_C1	0.372	0.587	0.570	No
CT_C2	0.427	0.565	0.576	Yes
CT_C3	0.467	0.537	0.569	Yes
CT_R1	0.419	0.573	0.573	Equal
CT_R2	0.417	0.561	0.566	Yes
CT_R3	0.448	0.535	0.546	Yes
CT_S1	0.352	0.584	0.599	Yes
CT_S2	0.370	0.577	0.585	Yes
CT_S3	0.362	0.554	0.572	Yes
CT_S4	0.349	0.529	0.563	Yes
PI_F1	0.267	0.631	0.660	Yes
PI_F2	0.388	0.614	0.629	Yes
PI_F3	0.304	0.621	0.656	Yes
PI_P1	0.441	0.619	0.645	Yes
PI_P2	0.513	0.592	0.599	Yes
PI_P3	0.394	0.565	0.593	Yes
PI_R1	0.343	0.597	0.611	Yes
PI_R2	0.238	0.654	0.678	Yes
PI_R3	0.334	0.624	0.647	Yes

Note. All Q^2predict values were positive. PLS-SEM showed lower RMSE than the linear-model benchmark for 17 of 19 indicators; CT_R1 was equal after rounding and CT_C1 favored the linear model.

Table S8. Latent-level PLSpredict and CVPAT results

Outcome	Q^2predict	RMSE	MAE	CVPAT ΔLoss vs IA	p	CVPAT ΔLoss vs LM	p
CT	.699	.553	.418	-.209	< .001	-.013	.020
PI	.669	.581	.433	-.216	< .001	-.028	< .001
Overall	—	—	—	-.213	< .001	-.020	< .001

Note. PLSpredict used 10 folds and 10 repetitions with a fixed seed. Negative CVPAT loss differences indicate lower prediction loss for PLS-SEM.

Table S9. Questionnaire item wording, construct allocation, consumer-trust subdimensions, and construct-level source basis

Original code	Final code	Construct	Trust subdimension	Item wording	Construct-level source basis
PV1	PQ1	Perceived Quality	—	Tottus dairy products are of good quality.	[10,11,16,19]
PV2	PQ2	Perceived Quality	—	Tottus dairy products maintain adequate quality over time.	[10,11,16,19]
PV3	PQ3	Perceived Quality	—	The packaging and visual presentation of Tottus dairy products are appropriate.	[10,11,16,19]
PV4	PQ4	Perceived Quality	—	Tottus dairy products have adequate freshness for household consumption.	[10,11,16,19]
PV5	EFV1	Economic-Financial Value	—	Tottus dairy products offer good value for money.	[10,11,16,19]
PV6	EFV2	Economic-Financial Value	—	I consider the price of Tottus dairy products to be reasonable.	[10,11,16,19]
PV7	EFV3	Economic-Financial Value	—	Buying Tottus dairy products is an economical option compared with other brands.	[10,11,16,19]
PV8	EFV4	Economic-Financial Value	—	I believe that the benefits of Tottus dairy products justify the price I pay.	[10,11,16,19]
PV9	FSV1	Functional-Sensory Value	—	Tottus dairy products are useful for my household's daily diet.	[10,11,16,19]
PV10	FSV2	Functional-Sensory Value	—	Tottus dairy products adequately meet my consumption needs.	[10,11,16,19]
PV11	FSV3	Functional-Sensory Value	—	Tottus dairy products have a pleasant taste and texture.	[10,11,16,19]
PV12	FSV4	Functional-Sensory Value	—	Tottus dairy products are suitable for my family's regular consumption.	[10,11,16,19]
PV13	EV1	Emotional Value	—	I enjoy buying Tottus dairy products.	[10,11,16,19]
PV14	EV2	Emotional Value	—	Consuming Tottus dairy products gives me a positive feeling.	[10,11,16,19]
PV15	EV3	Emotional Value	—	Buying Tottus dairy products provides me with a positive shopping experience.	[10,11,16,19]
PV16	SV1	Social Value	—	People close to me would consider it acceptable for me to buy Tottus dairy products.	[10,11,16,19]
PV17	SV2	Social Value	—	Buying Tottus dairy products would be viewed favorably by people in my family or social circle.	[10,11,16,19]
PV18	SV3	Social Value	—	Other people's opinions positively influence my willingness to buy Tottus dairy products.	[10,11,16,19]

CC1	CT_C1	Consumer Trust	Credibility	I consider Tottus to be a trustworthy brand in the dairy product category.	[15–17,19]
CC2	CT_C2	Consumer Trust	Credibility	I believe that Tottus delivers on what it promises regarding its dairy products.	[15–17,19]
CC3	CT_C3	Consumer Trust	Credibility	The information provided on Tottus dairy products inspires trust in me.	[15–17,19]
CC4	CT_R1	Consumer Trust	Reliability	I trust that Tottus dairy products maintain consistent quality.	[15–17,19]
CC5	CT_R2	Consumer Trust	Reliability	I trust that Tottus dairy products meet my consumption expectations.	[15–17,19]
CC6	CT_R3	Consumer Trust	Reliability	I trust that Tottus dairy products are suitable for my household.	[15–17,19]
CC7	CT_S1	Consumer Trust	Perceived safety	I feel safe consuming Tottus dairy products.	[15–17,19]
CC8	CT_S2	Consumer Trust	Perceived safety	I trust the origin and quality control of Tottus dairy products.	[15–17,19]
CC9	CT_S3	Consumer Trust	Perceived safety	I trust that Tottus dairy products are stored and marketed under appropriate conditions.	[15–17,19]
CC10	CT_S4	Consumer Trust	Perceived safety	I believe that buying Tottus dairy products does not pose a risk to my family.	[15–17,19]
IC1	PI_F1	Purchase Intention	—	I intend to buy Tottus dairy products on a future occasion.	[16,17,19,37]
IC2	PI_F2	Purchase Intention	—	I am likely to buy Tottus dairy products again.	[16,17,19,37]
IC3	PI_F3	Purchase Intention	—	I would consider including Tottus dairy products in my regular purchases.	[16,17,19,37]
IC4	PI_P1	Purchase Intention	—	I would consider choosing Tottus dairy products over traditional brands.	[16,17,19,37]
IC5	PI_P2	Purchase Intention	—	Tottus dairy products could become one of my main purchase options.	[16,17,19,37]
IC6	PI_P3	Purchase Intention	—	I would prefer to buy Tottus dairy products if I perceive that they offer good value.	[16,17,19,37]
IC7	PI_R1	Purchase Intention	—	I would recommend Tottus dairy products to family or friends.	[16,17,19,37]
IC8	PI_R2	Purchase Intention	—	I would speak positively about Tottus dairy products if my experience were good.	[16,17,19,37]
IC9	PI_R3	Purchase Intention	—	I would encourage other people to try Tottus dairy products.	[16,17,19,37]

Note. The questionnaire was administered in Spanish. The item wording shown here is the English reporting version used for the manuscript and supplement. Source references are reported at the construct level because the study combined and contextually

adapted previously published scales rather than claiming one-to-one provenance for every item. Consumer-trust subdimension allocation reflects the documented credibility (CC1–CC3), reliability (CC4–CC6), and perceived-safety (CC7–CC10) structure. All 37 indicators were retained in the revised primary model.
